# Local Anaesthesia Suppressing Idiopathic Ventricular Tachycardia: - A Cause of Non-inducible Arrhythmia During Electrophysiology Study

**DOI:** 10.1016/s0972-6292(16)30693-3

**Published:** 2013-11-15

**Authors:** Shomu Bohora, Vishal Poptani, Yash Lokhandwala

**Affiliations:** 1 U.N.Mehta Institute of Cardiology and Research Centre, Ahmedabad; 2 Arrhythmia Associates, Mumbai

**Keywords:** Idiopathic Ventricular Tachycardia, Lignocaine, Electrophysiology study

## Abstract

A 13 year old boy having idiopathic ventricular tachycardia had non-inducible tachycardia twice on electrophysiology (EP) study due to suppression of arrhythmia by local anaesthetic agent, lignocaine. This case report demonstrates a cause of non-inducibility or arrhythmia during EP study and effect of lignocaine in suppression of idiopathic ventricular tachycardia.

## Case Report

**Figure 1:**
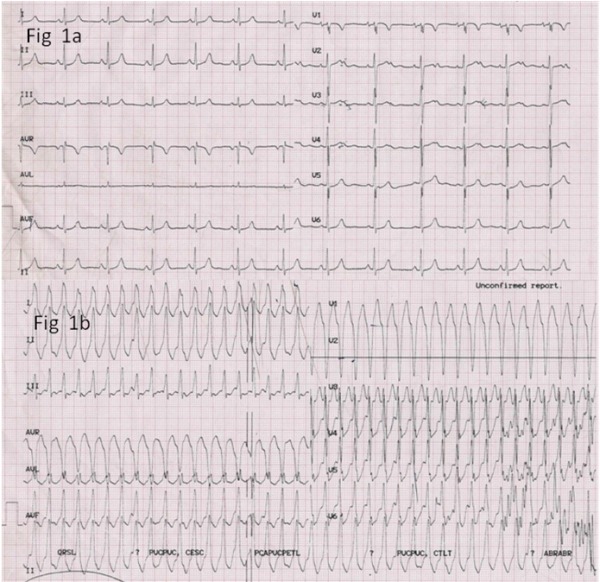
a. Sinus rhythm ECG. T wave notching in V2-4. Figure 1b. ECG during tachycardia showing a wide QRS tachycardia with LBBB and normal axis.

A 13 year old boy presented with complaints of intermittent fast palpitations of 3 months duration. The sinus rhythm ECG showed notching of T waves in V2-4 but was otherwise normal (Figure 1a). The ECG during tachycardia suggested a ventricular tachycardia (VT) with left bundle branch block (LBBB) morphology (Figure 1b) which was responsive to intravenous diltiazem and lignocaine when given at different occasions elsewhere. Even on amiodarone and diltiazem at maximum tolerated doses a 24 hour Holter showed presence of several runs of non-sustained VT (NSVT), though at slower rates and multiple premature ventricular complexes (PVCs). The echocardiogram was normal. Hence radiofrequency ablation was planned. After sedation with midazolam 1 mg and catheter placement there were no ectopics, NSVT or VT either spontaneously or on pacing protocols, even after isoprenaline. After four hours of observation, NSVT and PVCs reappeared.

**Figure 2:**
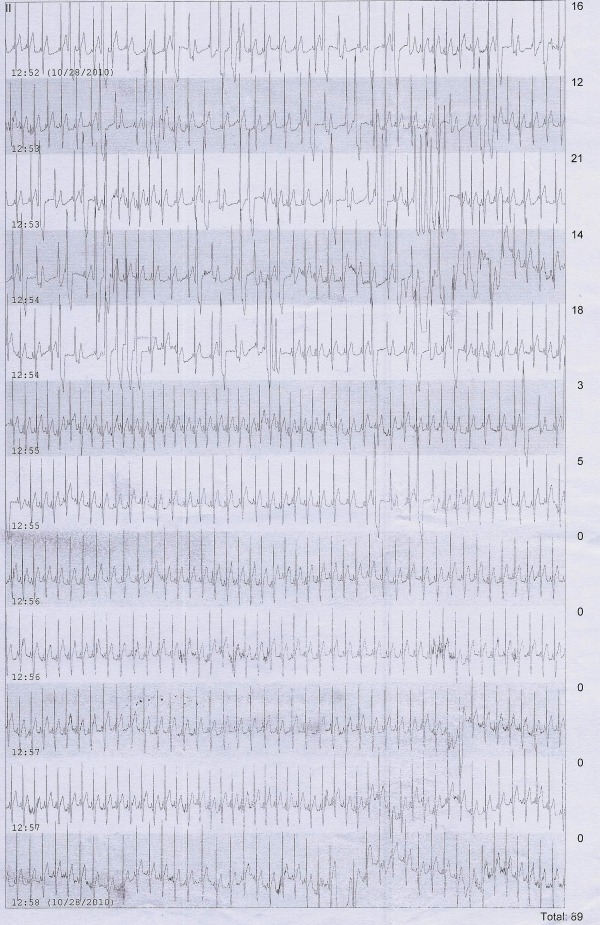
ECG recording after giving subcutaneous lignocaine in the ICU at 12:54 pm. No PVCs noted after 12:56 pm onwards. The later half of the recording does not show even a single PVC, which were very frequent along with NSVTs prior to subcutaneous lignocaine.

Considering non-inducibility of arrhythmia due to sedation, a restudy without sedation was planned. There were frequent PVCs and NSVT just prior to the study. However again after preparation and giving local anaesthesia, not even a single PVC was seen. That is when we realized that the patient's arrhythmia could have been extremely sensitive to lignocaine which was given subcutaneously as a local anesthetic agent. After 4 hours again the PVCs reappeared. To confirm our hypothesis, subcutaneous lignocaine (local anaesthetic) was administered in cardiac care unit (CCU). This suppressed the PVCs within 2 minutes (Figure 2) and the effect lasted for 4 hours. Subcutaneous bupivacaine also suppressed the PVCs for nearly 6 hours.

**Figure 3:**
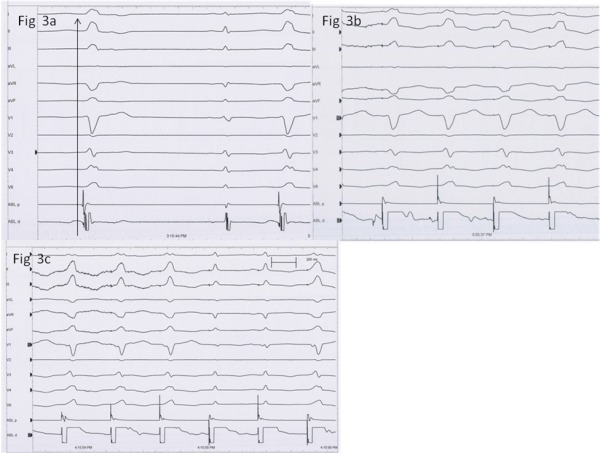
Electrophysiology study findings. Figure 3a. Activation mapping during spontaneous ventricular ectopics. Mapping signals 12 msec early than the surface QRS. Figure 3b. Pacemapping 12/12 match. Figure 3c During pacing from the same site, narrowing of the QRS suggesting His capture during respiratory movements.

Restudy was undertaken without giving local anesthetic; only sedation with 2 mg midazolam intravenously was used. A single femoral venous puncture was taken considering VT morphology, and an ablation catheter was used to map during NSVT and PVCs, which this time, did not get suppressed. The site of earliest activation was at a site near the His bundle (Figure 3a). Pace-map showed a 12/12 match at the same site (Figure 3b). Continued pacing caused intermittent His bundle capture with a narrowed QRS (Figure 3c). Ablation was hence not considered safe. A trial of intravenous phenytoin and metoprolol did not result in any decrease in the ectopics.

The patient still continues to have NSVT and VT even after giving diltiazem, though at a lesser frequency and rate, which respond immediately to lignocaine. The patient and relatives have been instructed to administer subcutaneous lignocaine in case of emergency and to consider ablation if significant symptoms persist, but with high chances of developing complete atrioventricular block during procedure.

## Discussion

Idiopathic VT was initially characterized as of right ventricular outflow origin or of left posterior fascicular origin. Increasingly over the years, it has been realized that idiopathic VT can be ablated at many other sites in the LV, RV, aortic cusps and pulmonary artery. Idiopathic VT has been treated by radiofrequency ablation with high success. Occasional patients fail to have tachycardia induction during the study despite all efforts. The mechanism in these patients is presumably automaticity or triggered activity. Idiopathic ventricular tachycardia has been responsive to various drugs including adenosine, beta-blockers, calcium channel blockers and class III antiarrhythmic drugs. Lignocaine has been used as an antiarrhythmic agent especially for ventricular tachycardia during ischemic settings.

This case report highlights the cause of non-inducibility of idiopathic VT on electrophysiology study, which occasionally has been attributed to general anesthesia or sedation. Lignocaine and bupivacaine have been commonly used local anaesthetic agents. Here we demonstrate a unique problem in a young patient with idiopathic ventricular tachycardia wherein the arrhythmia becomes completely non-inducible when given local anaesthesia. The arrhythmia subsided with intravenous lignocaine previously and that possibly could have been a clue when the arrhythmia did not occur during the 1st study. The total amount of lignocaine which was used in giving local anesthesia of 5 ml was 100 mg. This amount immediately suppressed the arrhythmia and maintained it for around 4 hours due to dual curve of lignocaine pharmacokinetics along with possible delayed subcutaneous release. The fact that phenytoin had no effect on suppressing the arrhythmia (though mexelitine could not be administered as it was not available) suggests lignocaine's effect to be non Class Ib antiarrhythmic effect. Bupivacaine also suppressed the arrhythmia, suggesting a class effect of local anaesthetic agents. Local anesthetics are membrane stabilizing drugs and they reversibly decrease the rate of depolarization and repolarization of excitable membranes, acting mainly by inhibiting sodium influx through sodium-specific ion channels in the cell membrane. Ventricular arrhythmias due to acceleration of ectopic foci may be responsive to lignocaine because of its effect on decreasing automaticity by slowing the rate of spontaneous phase 4 depolarization. Lignocaine has been shown to abolish the gating function of distal Purkinje tissue by reducing the non-uniformity of the action potential duration in Purkinje tissue, resulting in more uniform recovery of excitability, and to abolish slowing of conduction in Purkinje tissue. Reentrant ventricular arrhythmias may thus also be abolished by lidocaine, due to its effect on action potential durations resulting in altered conduction velocity and excitability. [1,2]

A restudy and mapping in the left ventricles/aorta and with attempt at ablation has been suggested in case the arrhythmia significantly affects the quality of life or causes tachycardiomyopathy. Cryoablation, which is not available, could have been a helpful. The case highlights that idiopathic ventricular tachycardia can be exquisitely sensitive to lignocaine and possibly it may be the cause of non-inducibility of arrhythmia during occasional electrophysiology study.
